# Motor Skill Acquisition and Retention after Somatosensory Electrical Stimulation in Healthy Humans

**DOI:** 10.3389/fnhum.2016.00115

**Published:** 2016-03-16

**Authors:** Menno P. Veldman, Inge Zijdewind, Nicola A. Maffiuletti, Tibor Hortobágyi

**Affiliations:** ^1^Center for Human Movement Sciences, University Medical Center Groningen, University of GroningenGroningen, Netherlands; ^2^Department of Neuroscience, University Medical Center Groningen, University of GroningenGroningen, Netherlands; ^3^Human Performance Lab, Schulthess ClinicZurich, Switzerland

**Keywords:** motor learning, motor memory consolidation, primary motor cortex, transcranial magnetic stimulation, motor evoked potential

## Abstract

Somatosensory electrical stimulation (SES) can increase motor performance, presumably through a modulation of neuronal excitability. Because the effects of SES can outlast the period of stimulation, we examined the possibility that SES can also enhance the retention of motor performance, motor memory consolidation, after 24 h (Day 2) and 7 days (Day 7), that such effects would be scaled by SES duration, and that such effects were mediated by changes in aspects of corticospinal excitability, short-interval intracortical inhibition (SICI), and intracortical facilitation (ICF). Healthy young adults (*n* = 40) received either 20 (SES-20), 40 (SES-40), or 60 min (SES-60) of real SES, or sham SES (SES-0). The results showed SES-20 increased visuomotor performance on Day 2 (15%) and Day 7 (17%) and SES-60 increased visuomotor performance on Day 7 (11%; all *p* < 0.05) compared with SES-0. Specific responses to transcranial magnetic stimulation (TMS) increased immediately after SES (*p* < 0.05) but not on Days 2 and 7. In addition, changes in behavioral and neurophysiological parameters did not correlate, suggesting that paths and structures other than the ones TMS can assay must be (also) involved in the increases in visuomotor performance after SES. As examined in the present study, low-intensity peripheral electrical nerve stimulation did not have acute effects on healthy adults' visuomotor performance but SES had delayed effects in the form of enhanced motor memory consolidation that were not scaled by the duration of SES.

## Introduction

Sensory input is critical for accurate motor performance. In addition, impaired sensory input decreases motor function in monkeys (Pavlides et al., 1993) and humans (Gentilucci et al., 1997), inevitably contributing to a variety of movement disorders (Patel et al., 2014). At the segmental level, spinal interneurons act as integrators between the sensory input and motor output (Nielsen, 2004). At the cortical level, there is a strong interaction between afference and efference through direct paths interconnecting the somatosensory cortices and the primary motor cortex (M1) in rodents (Manita et al., 2015) and humans (Jones, 1983). Unsurprisingly, manipulation of sensory input is widely used in motor learning and movement rehabilitation, for example, following a stroke (Wu et al., 2006; Conforto et al., 2007).

The effects sensory inputs can exert on motor function are exploited by the idea that non-physiological sensory input could increase motor performance. Such improvements in motor performance could be mediated by increases in activation of somatosensory and motor cortices after sensory inputs in the form of somatosensory electrical stimulation (SES) (Golaszewski et al., 2004; Wu et al., 2005) and long-term potentiation (LTP)-like mechanisms. Indeed, transcranial magnetic stimulation (TMS) studies reported increases in corticospinal excitability (Ridding et al., 2001; Kaelin-Lang et al., 2002; Andrews et al., 2013), increases in intracortical facilitation (ICF) (Kobayashi et al., 2003), and decreases in intracortical inhibition (Classen et al., 2000) after SES. The increases in M1 activity and excitability are suggested to originate in S1 through LTP-like mechanisms, indicated by correlated increases in primary motor and sensory cortex excitability (Schabrun et al., 2012), changes that are essential for skill acquisition and retention (Pavlides et al., 1993; Cantarero et al., 2013). Collectively, these studies provide a neuroanatomical and neurophysiological basis for how SES can increase motor performance.

SES targeting cutaneous and muscle afferents of peripheral nerves can enhance visuomotor and functional skill acquisition in healthy individuals (Veldman et al., 2015) and stroke patients (Wu et al., 2006; Celnik et al., 2007; Conforto et al., 2007; Koesler et al., 2009). In addition, stroke patients can consolidate these acquired skills into motor memory 24 h (Celnik et al., 2007) and 30 days (Conforto et al., 2007) after SES. Motor memory consolidation is a process that is observed as long as 8 years after motor practice (Brown et al., 2009; Borich and Kimberley, 2011; Park et al., 2013) and therefore relevant for rehabilitation practice. While M1 is suggested to be one of the key regions in motor skill acquisition, motor memory consolidation seems to rely on a more extensive network of brain areas, including M1, S1, parietal, and striatum-cerebellar networks (Dayan and Cohen, 2011). Imaging data show increased activity in premotor, posterior parietal, and cerebellar regions after SES; areas that are, directly or indirectly, connected to M1 (Forss et al., 1994; Wu et al., 2005; Manto et al., 2006). Because these areas are relevant for consolidation of motor memories (Shadmehr and Holcomb, 1997), it is possible that SES can enhance motor memory consolidation. However, to the best of our knowledge it is not known whether these off-line consolidation effects are also present in a healthy population and whether such effects are partly, if at all, mediated by changes in neuronal excitability.

Despite the available and promising data, the variability between studies concerning the SES-induced effects on motor performance and corticospinal excitability is high, possibly caused by differences in the stimulation parameters used. For example, different intensities and frequencies of SES can have opposite effects on motor performance and cortical excitability (Chipchase et al., 2011; Veldman et al., 2014). Specifically, SES at or below 10 Hz consistently increases both motor performance and corticospinal excitability (Veldman et al., 2014) while higher stimulation frequencies seem to decrease corticospinal excitability (Schabrun et al., 2012). Furthermore, SES intensities just below motor threshold tends to increase motor performance more consistently compared to lower (perceptual threshold) or higher intensities (above motor threshold) while opposite effects are observed in the SES-induced effects on corticospinal excitability (Veldman et al., 2014).

While frequency and intensities are important parameters, the duration of SES is also expected to play a role in motor adaptations. However, a systematic examination of the effects of SES duration on motor performance and corticospinal excitability is lacking. The limited data on SES duration suggest that SES for 40 compared with 60–120 min is sufficient to produce maximal increases in corticospinal excitability (McKay et al., 2002; Andrews et al., 2013). Clinical studies consistently used, for unspecified reasons, 120 min of SES to increase stroke patients' motor performance (Wu et al., 2006; Celnik et al., 2007; Conforto et al., 2007; Koesler et al., 2009). However, the excitability increases of cutaneous afferents after trains of electrical stimuli with 7–12 min duration last only 10 min (Applegate and Burke, 1989). In addition, skill acquisition and motor memory consolidation are associated with LTP (Cantarero et al., 2013), which increases field potential amplitudes 25–35 min after induction of LTP by theta-burst stimulation (Hess and Donoghue, 1994). In addition, increases in corticospinal excitability after SES are observed starting from 15 min after the onset of SES (McKay et al., 2002). These data provide a hint that 20 min of SES may already be sufficient to produce skill acquisition and motor memory consolidation.

To address the aforementioned issues, the aim of the present study was to examine the acute and delayed effects of SES applied for 20, 40, or 60 min on motor performance in a visuomotor task in healthy young adults. Additionally, we aimed to gain insights into the neuronal mechanisms underlying the acute and delayed effects of SES by quantifying corticospinal excitability, short-interval intracortical inhibition (SICI), and ICF before, immediately after, 24 h after (Day 2), and 7 days (Day 7) after the interventions.

## Materials and methods

### Participants

Forty healthy right-handed adult volunteers participated in this study. Before inclusion, we determined handedness (Oldfield, 1971) and the presence of any contraindications for the use of TMS through a health questionnaire (Rossi et al., 2009). All participants signed a written informed consent before participation; the study protocol was conducted according to the declaration of Helsinki and was approved by the Medical Ethical Committee of the University Medical Center Groningen.

### Experimental design

**Figure 1:**
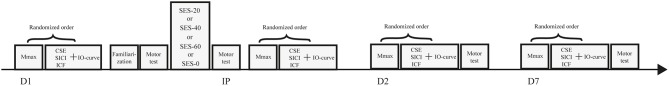
**Schematic overview of the experimental design**. Baseline measures including maximal compound action potentials (Mmax), corticospinal excitability (CSE), short-interval intracortical inhibition (SICI), intracortical facilitation (ICF), and input-output curves (IO curve) were performed before familiarization of the visuomotor task and after completion of one of four somatosensory electrical stimulation (SES) interventions. Baseline measures were repeated immediately post SES (IP), on day 2 (D2), and on day 7 (D7).

After inclusion, participants were randomly assigned to one of the three intervention groups receiving either 20 (SES-20, *n* = 10, 4 men, 23 ± 2 y, 1.78 m, 73 kg), 40 (SES-40, *n* = 10, 6 men, 23 ± 3 y, 1.81 m, 78 kg), or 60 min of SES (SES-60, *n* = 10, 4 men, 22 ± 2 y, 1.75 m, 71 kg). Ten participants were assigned to a control group, and completed sham SES (SES-0, 4 men, 22 ± 2y, 1.77 m, 76 kg). An active control group controlling for spatial specificity was not included because the spatial specific nature of SES has already been shown in patients (Wu et al., 2006) and healthy participants (Koesler et al., 2008). Each participant visited the lab on three different occasions and received only one intervention because consolidation of motor memory was expected. On the first day, baseline measures were taken using TMS and peripheral nerve stimulation. Next, participants were familiarized with the visuomotor task before baseline visuomotor performance was determined. Immediately, 24 h (Day 2), and 7 days (Day 7) after the intervention, baseline measures were repeated to determine acute and consolidation effects, respectively. Participants performed the follow-up tests at the same time (±2 h) relative to baseline to minimize circadian effects on SES-induced cortical plasticity (Sale et al., 2007). In addition, the quality and quantity of sleep over the experimental 1-week-period was determined using the Pittsburgh Sleep Quality Index (Buysse et al., 1989). Figure 1 depicts a schematic overview of the experimental design.

### Visuomotor testing

Participants sat in front of a laptop's computer monitor (diagonal dimension, 34 cm) in a chair without armrests. The left arm was resting on a table and the right hand was placed half-supinated in a padded manipulandum that allowed only the right wrist to move, with the thumbs superior. The feet were flat on the ground with the knees flexed 90°.

Visuomotor performance was determined using 12 consecutive trials of a visuomotor tracking task. Participants followed a pre-programmed template as accurately as possible by flexing and extending the wrist in the transverse plane, which moved a cursor downwards and upwards, respectively, while the cursor progressed from left to right at a fixed speed that varied from 3.3 to 4.0 cm/s between trials. Visuomotor templates appeared in white over a sharp blue background. There were six different visuomotor templates that appeared in white over a sharp blue background and were presented to the participants in a random order. The order of visuomotor trials was similar each time behavioral performance was determined. The duration of each trial varied from four to 6 s with an average duration of 5 s. Because the trials directly followed each other, the total duration of the behavioral testing was 1 min.

### SES interventions

During SES, participants sat in a chair with both arms resting on the table. Two electrodes (ConMed Cleatrode, Ag/AgCl, Ref 1720-003, NY, USA) were affixed to the skin over the radial and median nerves ±2 cm proximal to the right elbow. Electrical square wave pulses (pulse width, 1 ms) were applied using a constant-current electrical stimulator (Digitimer, model DS7A, Welwyn Garden City, UK) in 0.5-s-trains consisting of 5 pulses delivered at a frequency of 10 Hz, followed by a 0.5-s phase with no stimulation (50% duty cycle). Pulses with 1 ms width predominantly activate cutaneous and proprioceptive fibers (Panizza et al., 1992). SES intensity was set at just below the motor threshold (3.2 ± 1.6 mA) and was determined as the highest intensity without a motor response and pain in the wrist flexor and extensor muscles, causing mild paresthesia in the right arm. Participants were seated in front of a screen and instructed not to move the right arm during SES while the electrical pulses were shown on the screen represented by squares. At 5-min intervals, participants performed a counting task similar to the serial 7-s task used in the mini mental state examination to control for attentional drift (Tombaugh and McIntyre, 1992).

### Control intervention

The experimental setup in the control group was identical to the setup during real SES intervention. Electrical pulses were visualized on the projection screen. However, invisible to the participant, the cable was unplugged from the stimulator. The duration of this sham SES was 20, 40, or 60 min and varied randomly between participants.

### EMG recording

The skin over the muscle belly of the right extensor carpi radialis muscle (ECR) was shaved, gently rubbed with fine sand paper, and cleaned with alcohol before 37 × 26 × 15 mm, 14 g, wireless, pre-amplified parallel-bar sensors were affixed to the skin with a four-slot adhesive interface to record surface electromyographic (EMG) activity (Trigno, Delsys Inc., Natick, MA, USA) during electrophysiological measures. The EMG signal was sampled at 4 kHz using data acquisition software (Power 1401 and Signal, Cambridge electronics Design, Cambridge, UK). The data were recorded with a 20–450 Hz bandwidth and amplified 909 times, with a channel noise less than 0.75 μV, and a common mode rejection ratio over 80 dB. The data were stored on a personal computer for off-line analysis.

### Transcranial magnetic stimulation

Two Magstim 200 magnetic stimulators (Magstim, Dyfed, UK), connected through a BiStim module, were used to evoke motor evoked potentials (MEPs) with a figure-of-eight-shaped magnetic coil (loop diameter, 9 cm). With the handle pointing backwards at ~45° away from the sagittal plane, the coil was placed over the left M1 at the optimal spot to evoke MEPs in the right ECR. The optimal spot was marked on a cloth cap worn by the participants to ensure consistent coil placement throughout the experiments. Next, the resting motor threshold (rMT) was determined as the nearest 1% of stimulator output at which MEPs of at least 50 μV were evoked in the right ECR in five out of ten consecutive stimuli (5-s interstimulus interval with 10% variation).

SICI and ICF were measured in one TMS run. Stimuli were delivered with 10% inter-pulse variation according to a previously-established protocol (Kujirai et al., 1993). With a subthreshold stimulus set at 80% of rMT and a suprathreshold stimulus set at 120% of rMT, SICI (*n* = 10) and ICF (*n* = 10) were evoked with intervals of 2 and 10 ms between the subthreshold and suprathreshold stimulus, respectively. There were at least 5 s (10% variation) between subsequent trials at a constant TMS intensity regardless of changes in excitability (Garry and Thomson, 2009).

In a separate TMS run, input-output properties of the corticospinal path were determined using an input-output curve (IO curve) in all but one participant, in which the rMT was too high to stimulate at sufficient intensities to create a reliable curve. IO curves were obtained by randomly applying 10 intensity levels ranging from 90 to 180% of rMT with eight stimuli at each intensity (5-s inter-stimulus interval with 10% variation).

### Peripheral electrical nerve stimulation

Maximal compound action potentials (Mmax) in the right ECR were evoked using square-wave electrical pulses (pulse width, 1 ms) applied to the radial nerve by means of the same stimulator used for SES. This was done to normalize MEPs by Mmax, thus enabling comparison of pre-, post-, and follow-up measurements. The intensity of the electrical pulses was progressively increased from 3 mA with 5 mA increments (5-s inter-stimulus interval) until a plateau in the M-wave peak-to-peak amplitude was observed.

### Data analysis

The vertical mean absolute deviation between cursor and the preprogrammed template (i.e., in the y-direction) was calculated for each of the 12 test trials using custom Matlab software (Mathworks, Natick, Massachusetts, USA, version 2014a). Per trial a mean deviation was calculated for a complete template. This value was then averaged for 12 trials to calculate an average per participant. Percentage differences between the average visuomotor performance at each time point were calculated to quantify motor skill acquisition and motor memory consolidation. In addition, net skill acquisition and net motor memory consolidation was calculated as the magnitude of learning in SES groups minus the magnitude of learning in the control group.

We quantified the peak-to-peak amplitude of MEPs. Trials were excluded when TMS did not elicit a motor response or when MEPs differed more than two standard deviations from the mean (4% of all MEPs in total). SICI and ICF were expressed as the ratio of the conditioned peak-to-peak MEP amplitude and the non-conditioned peak-to-peak MEP amplitude; higher values for SICI and ICF represent less inhibition and more facilitation, respectively.

IO curves were determined as mean MEP amplitudes at each intensity. Next, IO curve parameters were calculated using the Bolzmann equation (Equation 1) where evoked muscle responses (EMR) with increasing intensity (S) are subdivided into several components: maximal evoked muscle response (EMR_max_) is the plateau of the IO curves, S_50_ is the stimulation intensity required to elicit a MEP with 50% of the maximal amplitude, and K is the slope at S_50_ (Devanne et al., 1997).

(1) $$\begin{array}{r} EMR\left(S\right)=\frac{EMR_{max}}{1+exp\left[\frac{S_{50}\;-\;S}{K}\right]} \end{array}$$

EMR_max_ most likely reflects a balance of excitatory and inhibitory components in the corticospinal tract, and the slope of the IO curve indicates the size of the subliminal fringe. In addition, we calculated the area under the IO curve (AUC) as a global measure of the excitability because this parameter is determined by both the slope and the plateau value of the IO-relation.

### Statistical analysis

All data are reported as mean ± standard deviation. Analyses were performed on log-transformed data when normality was not confirmed by the Shapiro-Wilk test using SPSS (version 22.0). All variables are reported in their original, non-transformed, form and significance was set at *p* < 0.05.

Multilevel analysis was performed using MLwin (version 2.29). Multilevel analysis is robust to missing values and solves the assumption of sphericity associated with repeated measures of variance (Quene and van den Bergh, 2004). In total, 4% of the TMS data were missing. In addition, multilevel analysis can handle baseline differences between groups by allowing intercepts to vary between participants. Therefore, random intercept and slope models (Model 1) were constructed for performance and TMS variables in which Time of measurement (level 1) was nested within Participants (level 2). Subsequently, separate models were made for Group effects of stimulation in general (Stimulation: SES and SES-0; Model 2) or Group effects of stimulation duration (Duration: SES-0, SES-20, SES-40, and SES-60; Model 2). To both models Time effects (pre, Post, Day 2, and Day 7; Model 2) and Group (Duration or Stimulation) by Time interactions (Model 3) were added to examine main and interaction effects for each variable. Spearman correlation analysis was performed on non-transformed and non-normally distributed change scores in the complete sample to identify significant relationships. Additional Spearman correlation analysis was performed to examine whether SES-induced changes in visuomotor performance correlated with observed changes in neuronal excitability.

## Results

The four groups were similar in age, mass, and height, and had similar quantity and quality of sleep (Table 1).

**Table 1:** **Participant characteristics**.

	**Age (y)**	**Gender**	**BMI (kg/m^2^)**	**PSQI**	
				**D2**	**D7**
	**Mean (SD)**	**M/F**	**Mean (SD)**	**Mean (SD)**	**Mean (SD)**
SES-20	22.7 (2.3)	4/6	23.1 (3.1)	2.4 (1.5)	4.0 (2.3)
SES-40	22.5 (2.8)	6/4	23.5 (4.6)	2.4 (1.7)	2.9 (2.0)
SES-60	21.6 (1.5)	4/6	22.9 (2.1)	3.5 (2.1)	3.9 (2.3)
SES-0	21.5 (1.7)	4/6	23.8 (1.8)	3.6 (2.8)	4.1 (2.4)

*Participant characteristics are presented for each experimental group as mean ± SD. BMI, body mass index; PSQI, Pittsburgh Sleep Quality Index (lower scores represent higher quality of sleep); D2, Day 2; D7, Day 7*.

### Behavioral data

**Figure 2:**
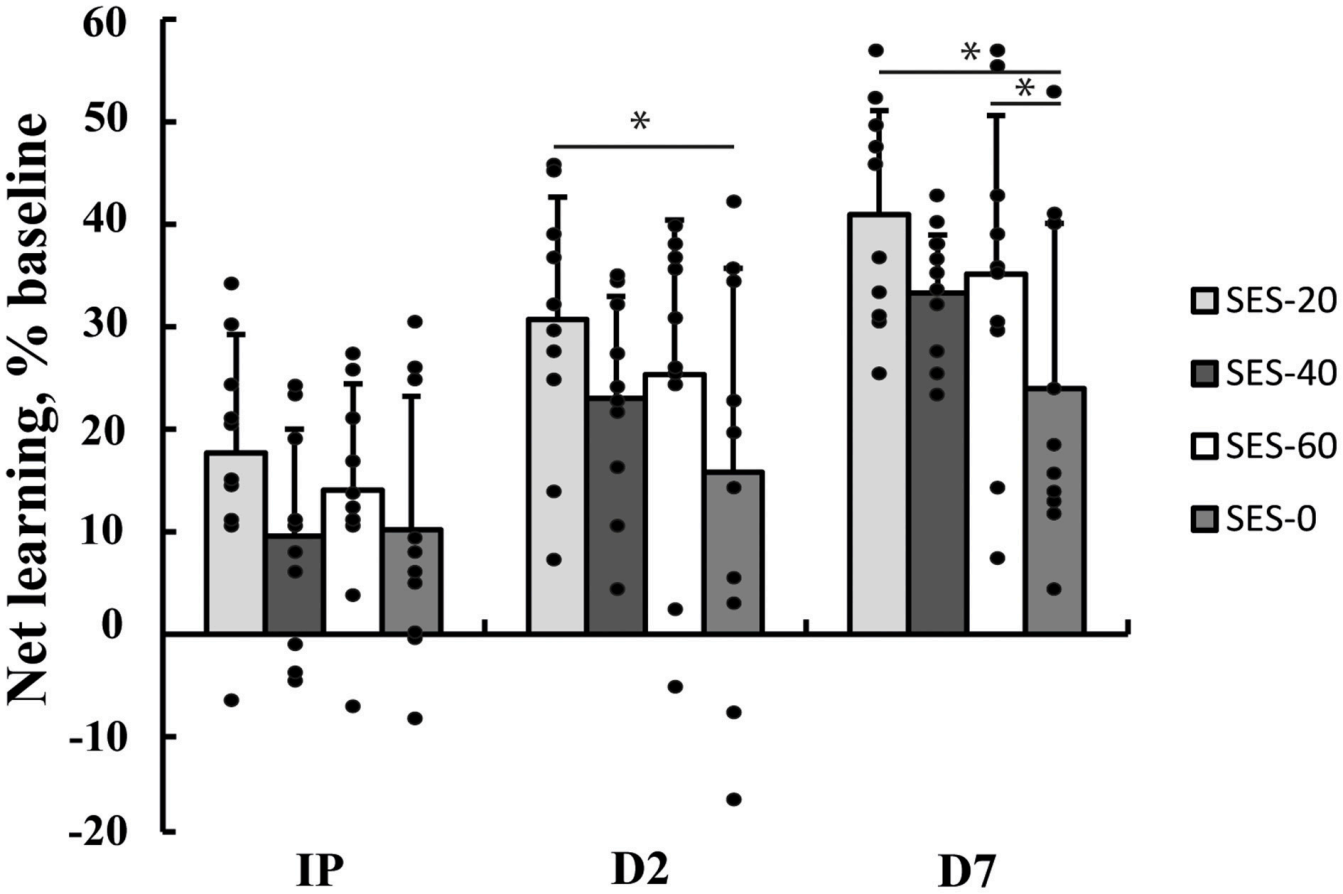
**Increases in visuomotor performance after 0, 20, 40, or 60 min of somatosensory electrical stimulation (SES)**. Percent increases are corrected for improvements as a result of familiarization with the task immediately post (IP), on Day 2 (D2), and Day 7 (D7). Performance increased more in SES-20 compared to SES-0 on Day 2. On Day 7, performance increased more in SES-20 and SES-60 compared to SES-0. ^*^Group by Time interaction with SES-0 (*p* < 0.05). Black dots represent individual changes. Vertical bars denote +1 SD.

Multilevel analysis showed that there was significant variability within (level 1) and between (level 2) participants. Specifically, the variance partition coefficient was 67% (*p* < 0.05), justifying the use of a multilevel model. Visuomotor performance increased significantly over Time (*p* < 0.05) in absence of an effect of Group (Stimulation and Duration; χ^2^ = 144.8, *p* < 0.001; Figure 2). The interaction effects significantly improved the model (χ^2^ = 20.2, *p* < 0.05) and showed that after SES-20, visuomotor performance increased on Day 2 (31% ± 12.0) and Day 7 (41% ± 10.1) relative to SES-0 (Day 2: 16% ± 19.9 and Day 7: 24% ± 16.1, both *p* < 0.001). Furthermore, visuomotor performance also increased after SES-60 on Day 7 (35% ± 15.5) compared to SES-0 (24% ± 16.1; *p* = 0.022). To reiterate, the multilevel analysis, that handled baseline differences by allowing intercepts to vary, revealed that there were delayed effects of SES on motor memory consolidation effects that were not proportional to the duration of SES. Table 2 summarizes the behavioral data.

**Table 2:** **Behavioral data**.

	**Pre** **Mean (SD)**	**IP** **Mean (SD)**	**D2** **Mean (SD)**	**D7** **Mean (SD)**
SES-20	20.5 (3.39)	16.8 (2.79)	14.0 (1.65)^*^	12.1 (2.73)^*^
SES-40	18.5 (3.25)	16.7 (3.10)	14.2 (2.95)	12.4 (2.42)
SES-60	17.7 (2.59)	15.2 (2.47)	13.7 (3.62)	11.3 (2.38)^*^
SES-mean	18.9 (3.08)	16.2 (2.79)	14.0 (2.74)	11.9 (2.51)^*^
SES-0	16.7 (4.48)	14.6 (2.80)	13.7 (3.66)	12.3 (2.82)

*Values are presented as mean ± SD for the pre, immediately post (IP), Day 2 (D2), and Day 7 (D7) measurement. The values represent deviation from the preprogrammed template in degrees (°). Somatosensory electrical stimulation (SES)-mean represents the average of SES-20, SES-40, and SES-60*.

* *p < 0.05 relative to SES-0 at IP*.

### Neuronal excitability

**Figure 3:**
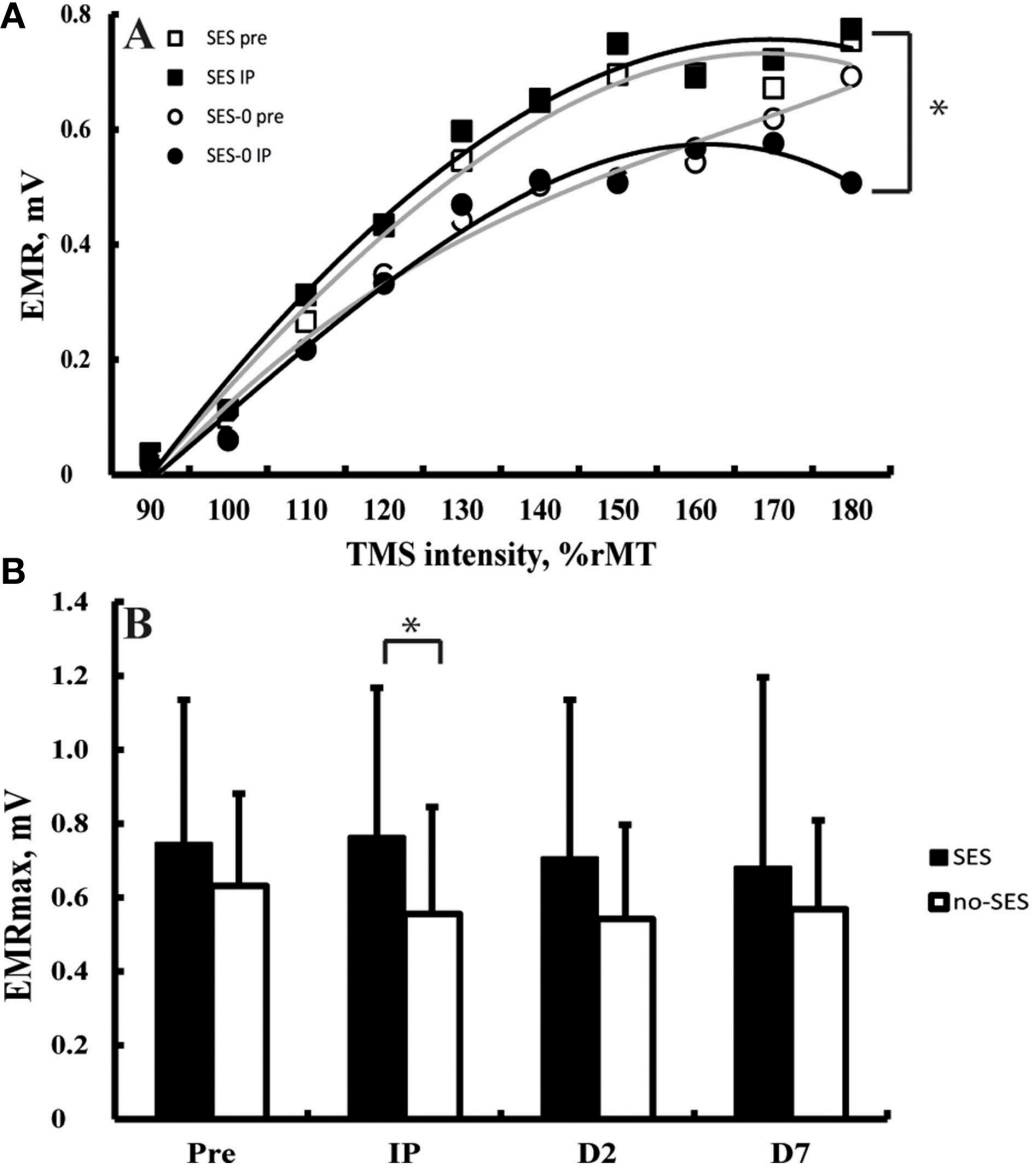
**(A)** Input-output curves before (equation: y = −0.0005x^3^ − 0.0031x^2^ + 0.1649x − 0.1628) and after (equation: y = −0.0002x^3^ − 0.0081x^2^ + 0.1946 − 0.1888) somatosensory electrical stimulation (SES) and before (equation: y = 0.0006x^3^ − 0.0155x^2^ + 0.1806 − 0.1819) and after (equation: y = −0.0009x^3^ + 0.0048x^2^ + 0.1084 − 0.1216) a control intervention. Panel **(B)** Maximal evoked motor responses (EMR_max_) increased after SES compared to SES-0 Immediately Post intervention (IP), but not on Day 2 (D2) and Day 7 (D7). ^*^Group by Time interaction (*p* < 0.05). Vertical bars denote + 1 SD.

All TMS metrics showed significant level-2 variation (range variance partition coefficient: 14–77%, all *p* < 0.05). There were no effects of Group (Stimulation and Duration), and Time on K, S_50_, and AUC computed from IO curves (all *p* > 0.05; Figure 3A). EMR_max_, however, was found to increase with borderline significance immediately after SES in the SES groups combined (5 ± 24%) compared to SES-0 (−19% ± 30.7; *p* = 0.02; χ^2^ = 9.6, *p* = 0.006; Figure 3B) but on Days 2 and 7, this was not significant anymore. In contrast to EMR_max_, SICI and ICF were not modified after SES. Table 3 summarizes the corticospinal and intracortical excitability data.

**Table 3:** **Transcranial magnetic stimulation data**.

		**Pre** **Mean (SD)**	**IP** **Mean (SD)**	**D2** **Mean (SD)**	**D7** **Mean (SD)**
EMR_max_	SES-mean	0.75 (0.39)	0.76 (0.40)^*^	0.71 (0.43)	0.68 (0.51)
	SES-0	0.63 (0.25)	0.55 (0.29)	0.54 (0.26)	0.57 (0.24)
AUC	SES-mean	44.5 (22.3)	46.8 (21.6)	41.5 (23.1)	42.8 (31.9)
	SES-0	36.0 (17.2)	35.0 (19.8)	32.7 (17.4)	34.3 (18.7)
SICI	SES-mean	56.4 (30.3)	58.3 (24.0)	49.6 (27.0)	59.3 (26.2)
	SES-0	56.9 (35.5)	40.5 (15.8)	46.8 (20.1)	46.5 (22.3)
ICF	SES-mean	142.4 (39.4)	142.1 (45.9)	129.8 (46.3)	140.3 (43.7)
	SES-0	127.9 (26.5)	120.7 (46.5)	125.7 (22.8)	132.2 (23.4)

*Values are presented as mean ± SD for the pre, immediately post (IP), Day 2 (D2), and Day 7 (D7) measurement. EMR_max_, maximal evoked motor response (mV); AUC, area under the curve (mV.maximal stimulator output); SICI: short-interval intracortical inhibition (% test pulse size); ICF, intracortical facilitation (% test pulse size). Somatosensory electrical stimulation (SES)-mean represents the average of SES-20, SES-40, and SES-60*.

* *p < 0.05 relative to SES-0 at Pre*.

### Correlation analyses

**Figure 4:**
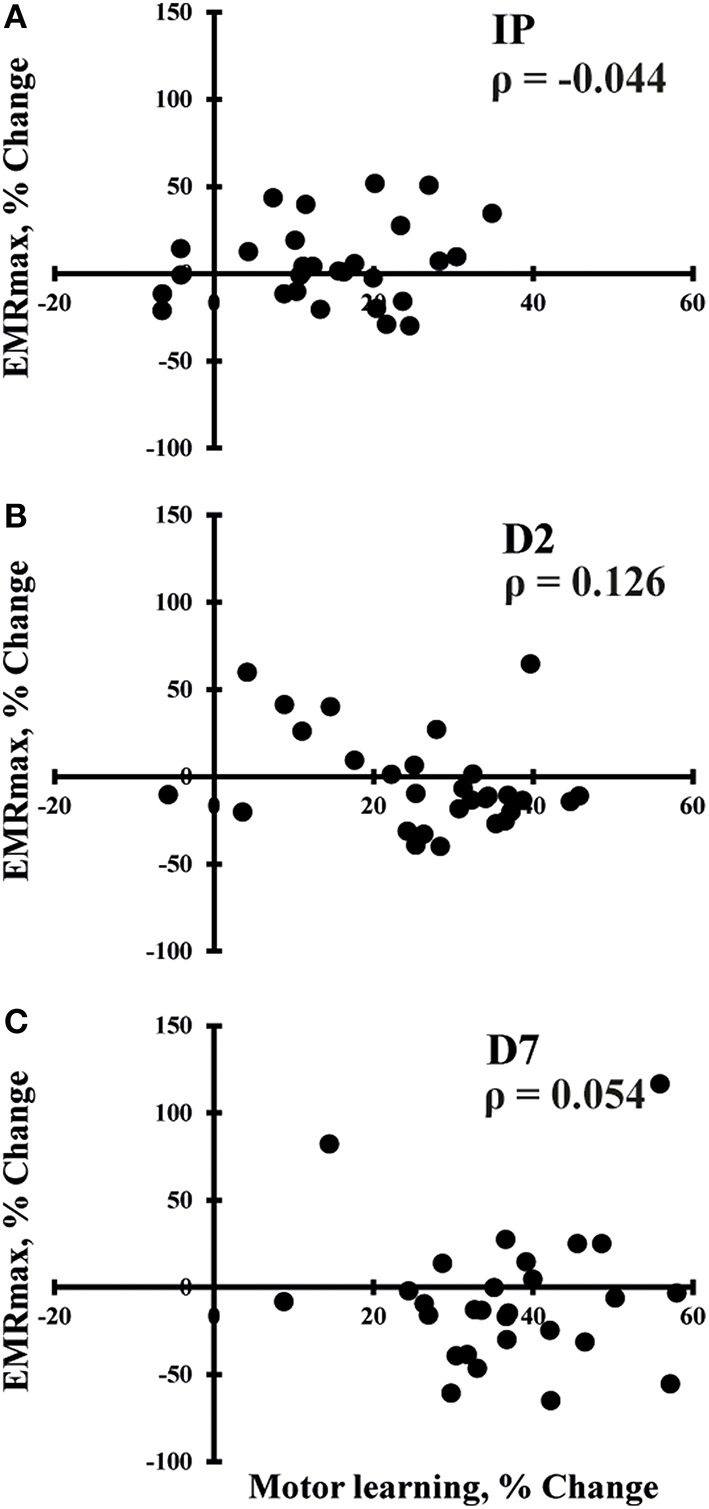
**Spearman correlations between changes in maximal evoked motor response (EMR_***max***_) and changes in motor performance immediately after SES (IP; ***n*** = 30; A), on day 2 (D2; ***n*** = 30; B), and on day 7 (D7; ***n*** = 30; C)**. No significant correlations were observed.

**Figure 5:**
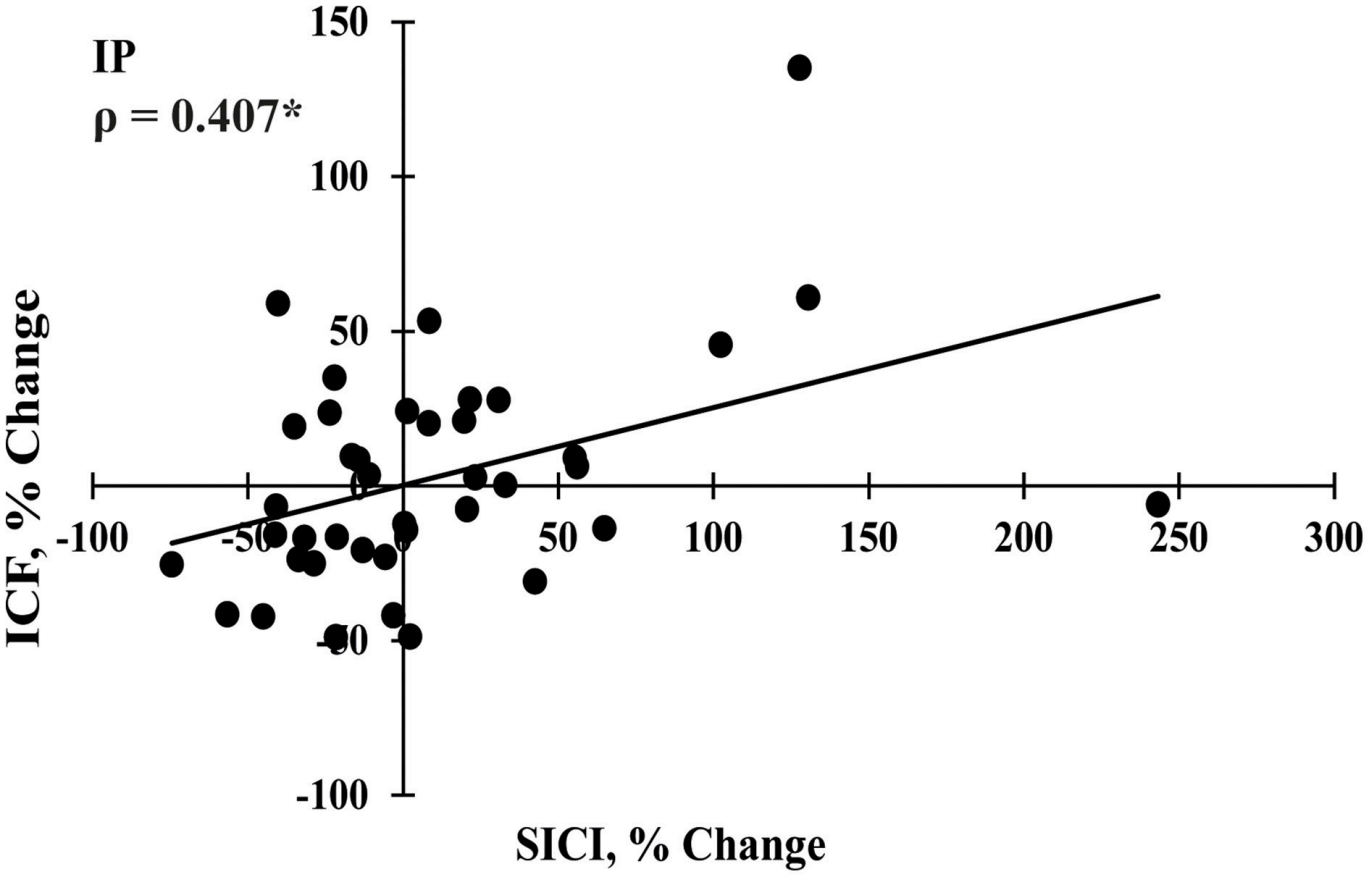
**Spearman correlation between changes in short interval intracortical inhibition (SICI) and intracortical facilitation (ICF) immediately post intervention (IP; ***n*** = 40)**. ^*^Significant correlation at *p* < 0.05.

SES-induced improvements in visuomotor performance did not correlate with changes in EMR_max_ immediately after SES (Figure 4A), on Day 2 (Figure 4B), and on Day 7 (Figure 4C), similar to the other neurophysiological parameters. However, changes in SICI and ICF observed after the interventions were moderately correlated (ρ = 0.407, *p* = 0.009; Figure 5), indicating that a decreased inhibition correlated with increased facilitation. These moderate correlations were absent on Days 2 and 7.

## Discussion

The present data show that SES did not improve visuomotor performance acquisition (4%) but in certain conditions, SES produced delayed effects on Day 2 (SES-20: 15%) and Day 7 (SES-20: 17%; SES-60: 11%) that were not proportional to SES duration. In contrast, specific responses to TMS increased only immediately after SES and were also not proportional to the duration of SES. Collectively, low-intensity peripheral electrical nerve stimulation did not acutely improve healthy adults' visuomotor performance but did produce delayed effects in the form of enhanced motor memory consolidation after SES that were not proportional to the duration of SES. We interpret these results from the perspective of how sensory inputs modulate motor output and the relationship between TMS metrics and motor output.

### Behavioral data

#### Acquisition effects

SES can improve healthy participants' motor performance, but these observations are inconsistent and the dose-response effects in terms of stimulation duration are unclear. SES for 120 min meaningfully improved healthy adults' functional performance by 12% in a functional performance battery (Sorinola et al., 2012) but produced no substantial changes in the kinematics of a reach-to-grasp movement (Koesler et al., 2008). Although 25 min of SES recently improved performance in a visuomotor task by 2.7° (6%; effect size: 1.24; *p* < 0.05) (Veldman et al., 2015), a similar paradigm produced smaller and non-significant changes in the present study (4%; effect size: 0.83; Figure 2). The source of these inconsistencies is unclear, but may be related to the intact state of the sensory and motor systems in the healthy participants because stroke patients in general, but especially those with more impairment, showed more prominent 15% improvements after SES (Conforto et al., 2002, 2007; Sawaki et al., 2006; Wu et al., 2006; Celnik et al., 2007; Koesler et al., 2009). Even though clinical studies used 120 min as “clinical standard,” these studies provided no physiological or clinical rational for delivering SES for this specific duration. To address this question, future studies will need to determine the dose-response relationship in terms of SES duration in patients to extend the present study conducted in healthy young adults.

The mechanism of how SES increases motor performance remains elusive. The sensory and motor systems interact through spinal interneurons at a segmental level (Nielsen, 2004)and through paths interconnecting the sensory and motor cortices, structures SES activates (Jones, 1983; Golaszewski et al., 2004; Wu et al., 2005; Manita et al., 2015). One possibility is that through these pathways, SES makes neurons in these areas more accessible to voluntary command. SES can expand cortical representations of the stimulated body parts in sensory and motor cortices (Golaszewski et al., 2002; Wu et al., 2005), and induces LTP-like plasticity indicated by increased corticospinal excitability (Ridding et al., 2001; Kaelin-Lang et al., 2002; Andrews et al., 2013), increased ICF (Kobayashi et al., 2003), and decreased GABAergically mediated SICI (Classen et al., 2000). GABA concentration is known to be associated with motor skill acquisition (Floyer-Lea et al., 2006) and pharmacologically enhanced GABA function blocks increases in corticospinal excitability (Kaelin-Lang et al., 2002) and use-dependent plasticity (Bütefisch et al., 2000). However, the low or altogether absent correlations between TMS metrics and behavioral outcome in the present and previous studies (e.g., Veldman et al., 2015) suggest that cortical and corticospinal excitability measures may contribute but are not directly related to motor performance. Especially for complex tasks, it is conceivable that neuronal processes, paths, and structures other than the ones TMS can assay are more involved in the SES-induced increases in motor performance. For example, synchronization of neural oscillations in the gamma/theta band within the parietal region (Perfetti et al., 2011) and beta coherence between M1 and parietal areas (Wu et al., 2014) has been shown to correlate with the magnitude of motor learning. Although beta coherence can predict motor cortex excitability outcome tested with TMS (Ferreri et al., 2014), TMS cannot fully capture functional connectivity between spatially distributed cortical areas.

#### Consolidation effects

Although SES did not produce enhanced skill acquisition, it produced effects on consolidation in the form of enhanced visuomotor performance on Day 2 (SES-20: 15%) and Day 7 (SES-20: 17%; SES-60: 11%) relative to SES-0. There was no dose-response relationship in the effects of SES on motor performance. Notwithstanding that the acute increases in corticospinal excitability were the greatest immediately after SES-40 (51%), the increases in visuomotor performance were not significant. Although the increases on Day 7 (9% relative to SES-0) did follow a similar pattern (Figure 2), the lack of significance may be related to the instability of the corticospinal drive after the greatest increases in corticospinal excitability immediately after SES-40 (Abbott and Nelson, 2000). These offline, between-session skill enhancements are a component of motor memory consolidation beyond stabilization (Robertson et al., 2004) and can last up to 8 years after motor practice (Park et al., 2013). The average motor memory consolidation after SES on Day 2 (10.6%) and Day 7 (12.5%) corresponds to healthy adults' motor memory consolidation following practice of a motor sequence (9.6%) (Brown et al., 2009) and tracking (10.8%) (Borich and Kimberley, 2011). Thus, SES can evoke delayed effects similar to the effects produced by motor practice without SES. The SES-effects tend to peak 24 h after the intervention in healthy participants. In stroke patients, SES seems to enhance motor memory consolidation further beyond these 24 h, an effect likely related to the impaired sensory and motor state having more room for improvement.

The M1 plays an important role in motor memory consolidation (Muellbacher et al., 2002; Baraduc et al., 2004). An occlusion of LTP in M1 interferes with skill consolidation (Cantarero et al., 2013) whereas anodal transcranial direct current stimulation over M1 enhances skill consolidation (Zimerman et al., 2013). SES may have enhanced motor memory consolidation by acting on M1 through direct connections between sensory and motor areas (Jones, 1983; Manita et al., 2015). Trains of repeated sensory stimuli are suggested to resemble deep proprioceptive physiological stimuli inducing lasting changes in somatosensory evoked potentials (Kaelin-Lang, 2008). Increases in somatosensory evoked potentials after peripheral nerve stimulation in humans (Schabrun et al., 2012) and repeated rhythmic whisker stimulation in mice confirm this suggestion (Mégevand et al., 2009). Hyperexcitability of ascending sensory axons occurs after SES trains with a duration of at least 7–12 min and these effects persist for 18 min following the stimulation period (Applegate and Burke, 1989; Kiernan et al., 1997). Recent neurophysiological studies (Andrews et al., 2013) and the present study confirm these findings showing changes within 20 min. These nerve excitability studies suggest that maximal increases in axonal excitability is reached after only 7 min, and may explain the absence of a dose-response relationship in the present study. Consistent with a previous report, associated changes in M1 excitability after SES did not reach significance on Days 2 and 7. In contrast, SES-induced shifts in cortical maps can be retained days after SES (Ridding et al., 2001). In line with this, SES expanded the cortical representation of stimulated body parts in the primary sensory cortex, M1, and premotor cortex, indicated by increases perfusion and blood-oxygen-level-dependent voxel count (Wu et al., 2005).

SES may also have enhanced motor memory consolidation by increasing activity in premotor, posterior parietal, and cerebellar regions (Forss et al., 1994; Wu et al., 2005; Manto et al., 2006). These regions are known to be involved in motor memory consolidation (Shadmehr and Holcomb, 1997) and movement related activity in these areas increases after SES (Wu et al., 2005). The premotor cortex is responsible for planning of intended movements (Schubotz and von Cramon, 2003), and is connected to the somatosensory cortex and higher order associative areas such as the parietal cortex (Cavada and Goldman-Rakic, 1989). We speculate that SES may have induced lasting representational reorganization in these areas and thereby augmented offline skill enhancement through increasing activity in these structures. In sum, the present study shows that SES can enhance motor memory consolidation in healthy adults, independent on stimulation duration. Such positive effects occurred in absence of correlations with changes in neuronal excitability, suggesting that mechanisms, paths, and/or structures other than those examined in the present experiment, such as the premotor, parietal, and cerebellar areas, may have been responsible for the observed consolidation effects. Although additional studies are needed to examine the exact time-course of induced changes in axonal excitability and associated activity and excitability, lasting changes in topographical maps after SES as a result of strengthened connections through Hebbian-like plasticity could underlie the enhanced motor memory consolidation observed in the present study.

### Neuronal excitability

#### Corticospinal excitability

In agreement with our hypothesis, SES at sensory intensities increased EMR_max_ without affecting the slope of the recruitment curve (5%; Figure 3B) (Kaelin-Lang et al., 2002; Khaslavskaia et al., 2002; Knash et al., 2003). Such effects were independent of SES duration. Increases in measures of corticospinal excitability after only 20 min of SES agree with previous findings (Andrews et al., 2013). High-frequency SES can make sensory axons hyperexcitable in 7–12 min, an effect that can outlast SES by up to 18 min (Applegate and Burke, 1989; Kiernan et al., 1997). The hyperexcitability of sensory axons can be due to high extracellular [K^+^] and pump-induced hyperpolarization (Kiernan et al., 1997). At a cortical level, the present observations roughly correspond with the time-course of induction of LTP after theta-burst stimulation (25–35 min after induction of LTP; Hess and Donoghue, 1994). However, considering the methodological differences between these and the present studies, the development of axonal excitability and LTP after low-frequency SES in healthy participants and patients over time periods up to 120 min needs to be clarified in future studies to provide insight into the time-course of induction of hyperexcitability at a peripheral level that could underlie changes in neuronal excitability and motor performance.

The data are also compatible with observations that SES at sensory intensities tends to increase recruitment curve plateaus indicating an increase in the maximal output of corticospinal neurons through changes in the balance of excitatory and inhibitory components in the corticospinal tract (Kaelin-Lang et al., 2002; Khaslavskaia et al., 2002; Knash et al., 2003). In contrast, SES at intensities sufficient to produce muscle twitches rather increases MEP amplitudes in the low-intensity portion of the recruitment curve (McKay et al., 2002; Knash et al., 2003; Andrews et al., 2013). These data indicate that SES at sensory intensities increases the maximal output of corticospinal neurons rather than increasing the excitability of the descending projections. Because the motor task in the present study only required low forces, increases in EMR_max_ may not have been relevant to the increases in visuomotor performance. The lack of correlations between increases in EMR_max_ and changes in motor performance (Figures 4A–C) reinforces this idea and suggest that increases in EMR_max_ rely on different neuronal populations than the ones responsible for visuomotor performance in the present study.

This suggestion is supported by our observations on Days 2 and 7. SES increased EMR_max_ acutely but not on Days 2 and 7, while the increases in motor performance after SES only became evident days after SES ended. Besides the order of measures (i.e., TMS measures before motor test on Days 2 and 7), we consider two other factors to explain a lack of correlations between behavioral and neurophysiological parameters. First, within 6 h after motor practice, brain activity shifts to prefrontal, parietal, and cerebellar regions (Shadmehr and Holcomb, 1997). Such a shift may explain why SES had no effects on TMS outcomes measured in M1 on Days 2 and 7. Second, error-based learning involves not only M1 but additional areas associated with motor planning, error detection and correction, working memory, and attention such as the basal ganglia thalamocortical loops, cerebellar areas, anterior cingulate cortex, inferior frontal gyrus, visual, and parietal areas (Hikosaka et al., 2002; Seidler and Noll, 2008; Seidler, 2010; Dayan and Cohen, 2011). Thus, it is not entirely surprising that the increase in visuomotor performance did not correlate with changes in TMS measures obtained in M1. It is possible that SES augmented retention compared with control by increasing the excitability of cortical structures within these regions. These increases in excitability subsequently could have made motor control more accurate on Days 2 and 7. Altogether, the present data show an increase in a specific aspect of corticospinal excitability that does not explain the observed skill enhancement on Days 2 and 7, a finding consistent with recent results (Bologna et al., 2015), indicating that neuronal excitability, as measured by TMS, is not necessarily related to behavioral outcome at retention.

#### Intracortical excitability

After SES, the decrease in SICI (20%) was not significant and did not correlate with immediate and delayed improvements in visuomotor skill on Days 2 and 7. Changes in GABA_A_-mediated SICI reflect LTP-like mechanisms in inhibitory horizontal connections (Hess and Donoghue, 1996) and tends to decrease after SES in stroke patients (Celnik et al., 2007). In contrast, SES seems not to affect SICI in healthy participants in previous (Kaelin-Lang et al., 2002; Veldman et al., 2015) and the present study. Similarly, ICF was also not modified after SES (6%), in agreement with data obtained in healthy participants (Kaelin-Lang et al., 2002; Veldman et al., 2015) and stroke patients (Celnik et al., 2007). Although the changes were not significant and did not correlate with increases in visuomotor performance, acute effects of SES on SICI and ICF did correlate with each other (Figure 5). These data suggest that SES may modulate the excitability of intracortical circuits and gives a hint, in contrast to previous suggestions (Kaelin-Lang et al., 2002), that LTP-like mechanisms contribute to neuronal and behavioral changes after SES.

## Limitations and conclusion

First, between-group differences at baseline complicate the interpretation of skill acquisition and motor memory consolidation, although multilevel analysis does in part handle these differences. Second, results from our healthy sample cannot be generalized to patients. However, since the effects of SES on skill acquisition are generally stronger in patients compared to healthy participants (Wu et al., 2006; Celnik et al., 2007; Conforto et al., 2007; Koesler et al., 2009), it is likely that SES can also enhance motor memory consolidation in patients. Third, we did not control whether sham and real SES were perceived differently by the participants. Additionally, although spatial specificity has previously been shown in both patients (Wu et al., 2006) and healthy participants (Koesler et al., 2008), our experimental design did not check whether the SES-induced effects observed in the present study are specific to the stimulated area. Fourth, we performed TMS measures only at rest in conjunction with a task that involved actual muscle contractions, making the interpretation of the data challenging, an issue recently discussed (Berghuis et al., 2015; Opie et al., 2015). Finally, we did not use neuronavigation equipment for TMS to ensure consistent coil placement across days. However, considering the almost numerically identical values for rMT on all three separate days, we argue that TMS measures are performed correctly and can be compared across days.

In conclusion, SES can enhance motor memory consolidation 24 h and 7 days after stimulation, independent of stimulation duration. In addition, SES has acute effects on certain measures of corticospinal excitability in healthy participants. The absence of correlations between neuronal excitability and motor memory consolidation could indicate that these two phenomena occur with a different timing or that other structures are also involved in mediating these effects. SES is known to activate premotor, parietal and cerebellar areas. However, not measured by TMS, these structures are known to be involved in motor memory consolidation and could have contributed to the increases in performance on Days 2 and 7. Collectively, low-intensity electrical peripheral nerve stimulation did not acutely affect healthy adults' visuomotor performance but instead SES produced delayed effects in the form of enhanced motor memory consolidation that were not proportional to the duration of SES.

## Author contributions

Study design: MV, IZ, NM, TH; Data acquisition: MV; Data analysis: MV, TH; Interpretation of data: MV, IZ, NM, TH; Drafting and revising: MV, IZ, NM, TH; Final approval: MV, IZ, NM, TH.

### Conflict of interest statement

The authors declare that the research was conducted in the absence of any commercial or financial relationships that could be construed as a potential conflict of interest.

## Abbreviations

AUC: area under the curve
ECR: extensor carpi radialis
EMG: electromyography
EMR_max_: maximal evoked muscle response
ICF: intracortical facilitation
IO curve: input-output curve
LTP: Long-term potentiation
MEP: motor evoked potential
rMT: resting motor threshold
SES: somatosensory electrical stimulation
SICI: short-interval intracortical inhibition
TMS: transcranial magnetic stimulation.
